# Conserving *Citrus* Diversity: From Vavilov’s Early Explorations to Genebanks around the World

**DOI:** 10.3390/plants12040814

**Published:** 2023-02-11

**Authors:** Gayle M. Volk, Frederick G. Gmitter, Robert R. Krueger

**Affiliations:** 1 USDA-ARS National Laboratory for Genetic Resources Preservation, 1111 S. Mason St., Fort Collins, CO 80521, USA; 2 Citrus Research and Education Center (CREC), Institute of Food and Agricultural Sciences (IFAS), University of Florida, Lake Alfred, FL 33850, USA; 3 USDA-ARS National Germplasm Repository for Citrus and Dates, 1060 Martin Luther King Blvd., Riverside, CA 92507, USA

**Keywords:** collections, ex situ conservation, diversity, global conservation strategy, plant genetic resources, survey

## Abstract

Citrus is among the most economically important fruit crops. Its vast species diversity and global production was observed by N.I. Vavilov during his international plant explorations from the early to mid-1900s. Currently, ex situ citrus collections located around the world conserve and protect citrus genetic resources, as revealed in a survey conducted in 2021. Responses were received from 43 collections in 27 countries, of which 35 provided data regarding collection composition, management practices, and security, as well as other information. The six largest citrus collections have between 1000 and 1735 accessions. The largest accession holdings are mandarins and sweet oranges, although all citrus fruit types are maintained: mandarin, sweet orange, lemon, pummelo, grapefruit, hybrids, lime, sour orange, citron, kumquat, papeda, finger lime, and crop wild relatives. Diseases pose significant threats to collections, though some collections are maintained in a clean-plant state as a result of intensive sanitation efforts. National and regional quarantine regulations often limit the export and import of citrus plants or propagative materials, thus limiting the availability of materials at an international level. Resources, both financial and human, are necessary to ensure the long-term safety and security of citrus collections on a global scale. Future efforts to develop citrus genebanking communities will provide opportunities for improved conservation, as well as collaborations and training.

## 1. Introduction

Citrus, including oranges (*Citrus* × *sinensis* (L.) Osbeck), lemons (*C.* × *limon* (L.) Osbeck), limes *(C. aurantiifolia* (Christman.) Swingle), pummelos (*C. maxima* (Burm.) Merr.), grapefruits (*C.* × *paradisi* Macfad.), mandarins (*C. reticulata* Blanco), and other fruits, are among the most widely grown fruit crops on a global scale. Citrus is produced in sutropical, semitropical, and tropical regions around the world, with most commercial production between 20° and 40° latitude in the Northern and Southern hemispheres. Oranges represent the largest global harvested area, global production (in tons), and value, followed by mandarins, lemons/limes, and pummelos/grapefruits [1]. 

According to Kruse [2], the taxonomically complex *Citrus* genus has between 16 and 162 described species. Although *Citrus* is the most widely cultivated genus within the Rutaceae, the fruits of *Clausena lansium* (Lour.) Skeels are consumed fresh in China and other areas; the leaves of *Bergera koenigii* L. are used in cooking in South Asia; the fruits of *Aegle marmelos* (L.) Corrêa are the basis of beverages consumed in South Asia; the fruits of *Microcitrus* spp. are consumed as “bush food” in Australia; and species such as *Murraya paniculata* (L.) Jack and *Triphasia trifolia* (Burm. F.) P. Wilson are grown as ornamentals [3].

Wild *Citrus* species native to China and Southeast Asia have contributed to the development of important commercial varieties and rootstocks [4,5]. For example, *Poncirus trifoliata* L. has been used as a parent in rootstock breeding and many rootstocks worldwide are either pure *P. trifoliata* or its hybrids with various *Citrus* species [3]. *Microcitrus* spp., Aurantioideae, a subfamily crop wild relative native to Oceania, was shown to be potentially useful for breeding citrus-like fruit that are tolerant of Huanglongbing (HLB) [6]. More recently, *P. trifoliata* has also been explored as a potential genetic resource for the breeding tolerance of HLB [7,8]; a cultivar exhibiting a strong tolerance of HLB that comes from the introgression of *P. trifoliata*, ‘US SunDragon’, has been released by the USDA [9]. Between 1916 and 1933, the Russian scientist, Nicolai Vavilov, traveled the world to better understand the relationships between plant species diversity and their origins [10]. His theory of the centers of origin for cultivated crop plants proposed that genetic diversity is highest at the center of origin for each plant species [11]. Vavilov encountered diverse *Citrus* taxa in his global explorations and described them in his writings. In 1916, Vavilov saw citrus in the Mazanderan Province of Iran. In 1926–1927, Vavilov encountered commercially produced Jaffa sweet oranges (*Citrus sinensis*) in Palestine and Trans-Jordania, as well as other kinds of citrus in Italy and Spain. In 1929, Vavilov explored the Xinjiang area of China, followed by Japan, Taiwan, and Korea. During his visit to Japan, Vavilov noted large land areas with mandarin (‘kan-kans’ and ‘unshu’ types) and sweet orange, as well as peach and quince [12]. In his travels to Taiwan, Vavilov visited a tropical research station in Kagi where he observed rubber trees, mango trees, mangosteen, and “original collections of tropical citrus fruits of gigantic proportions, the size of a human head”. In Taiwan, Vavilov met the Japanese citrus expert, Tyôzaburô Tanaka, who had a small station of citrus trees as well as an herbarium, from whom Vavilov received information about the endemic plants of China and Japan. Vavilov also observed grapefruit, orange, and lemon production in Brazil in 1933–1934 [12]. Vavilov collected sweet orange, sour orange (*C.* x *aurantium* L.), and lemon (*C.* x *limon* (L.) Burm.), during his explorations and imported them into the USSR [11]. Vavilov’s observations of citrus diversity led him to propose that citrus is native to three of his eight centers of diversity: (1) the Chinese center; (2) the Indian (including the Assam region) center; and (3) the Indo-Malayan (Indochina and the Malay Archipelago) center [13]. The presence of diverse citrus in these regions is undisputed, although more recent molecular evidence suggests that *Citrus* specifically originated in the region that covers parts of what are currently identified as the Yunnan Province of China, Northern Myanmar, and extreme Northeastern India [4]. 

**Figure 1:**
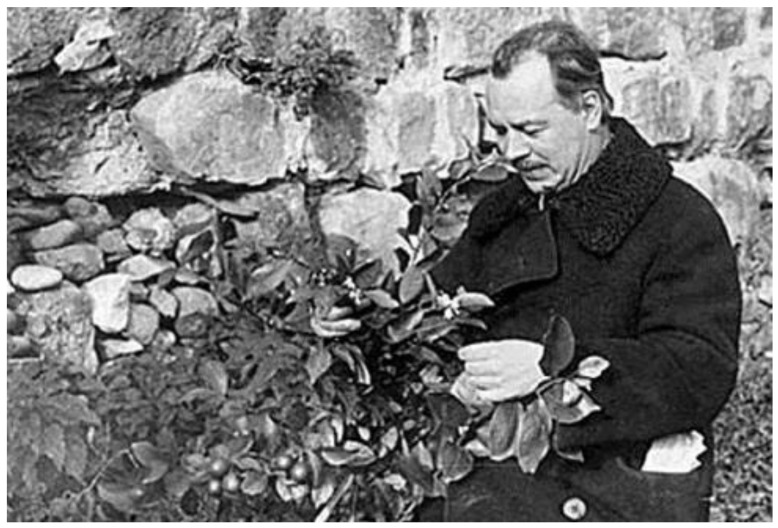
N.I. Vavilov inspecting citrus trees at Maykop, Russia, SFSR (a subdivision of the USSR) in 1935 (Novosti Press Agency).

Citrus has been produced in Russia since 1902. Frost tolerant citrus is grown along the Black Sea coast of the Western Caucasus, an area with an average maximum temperature of 28 °C and minimum temperature of 5.2 °C [14]. In 1914, the Black Sea coast region of Russia had 160 hectares in citrus (primarily mandarin) fruit production [15]. Georgia, which was incorporated into the Soviet Union as the Georgian Soviet Socialist Republic in 1922, produced significantly more citrus, with 3280 hectares under production in 1936. Vavilov was photographed in the region of Maykop, Russia, inspecting citrus trees in 1935 (Figure 1). 

The importance of plant genetic resource conservation has become widely recognized since the early explorations of Vavilov. Plant genebanks around the world conserve thousands of plant species, including *Citrus*. In addition to their conservation of valuable genetic resources, ex situ citrus collections are a critical source of novel diversity for breeding and research programs, particularly with the global threat of HLB, a disease apparently caused by the bacterium *Candidatus* Liberibacter asiaticus and spread by the Asian Citrus psyllid (*Diaphorina citri*) [16,17,18,19]. Additional diseases are associated with viruses (e.g., Citrus tristeza caused by *Closterovirus* Citrus tristeza virus [20]) and fungal/oomycete pathogens (e.g., *Phytophthora*-induced rots [21]) and also threaten citrus. Abiotic threats include salinity and other soil conditions, water availability and quality, and excessively hot or cold temperatures. 

We conducted a survey in 2021 to collect information about the status of citrus plant genetic resources as part of “A Global Strategy for the Conservation and Use of Citrus Genetic Resources”, a project coordinated by the Global Crop Diversity Trust. The survey identified both large and small citrus collections that conserve a wide range of genetic diversity [22]. 

## 2. Results

Citrus collections are maintained by traditional genebanks, public breeding programs, or by certification programs that produce and distribute high-quality planting materials free from systemic diseases. Certification programs are generally administered through a government entity, although operations may be delegated to industry-based organizations [23]. In some cases, a genebank might also have a certification component. More commonly, genebanks maintain a wide range of genetic resources, the most commercially useful of which the genebank can provide to certification programs, which in turn provide them to the industry. In some cases, the certification programs or genebanks propagate and sell trees. 

A total of 15,555 citrus accessions are maintained in the 35 collections for which inventory information was provided (Table S1). The largest collections are maintained by: the Instituto Agronômico de Campinas/Centro de Citricultura Sylvio Moreira, Brazil (1735 accessions); the Citrus Research Institute, Southwest University, China (1700 accessions); the USDA-ARS National Clonal Germplasm Repository for Citrus and Dates in the United States (1632 accessions); the Institute of Fruit Tree and Tea Science (NARO, NIFTS) in Japan (1261 accessions); the National Research Institute for Agriculture, Food and Environment (INRAE)-Corsica in France (1100 accessions); and the Queensland Department of Agriculture and Fisheries in Australia (1000 accessions). Additional collections, such as those in India, Laos, Nepal, Russia, South Africa, Spain, and Vietnam, include significant numbers of local cultivars and wild species materials. Ten collections that responded to the survey have fewer than 100 accessions (Table S1). The number of accessions recorded in citrus collections through this survey is more than four times the number of citrus accessions recorded in either the Genesys or FAO-WIEWS databases [24,25].

**Figure 2:**
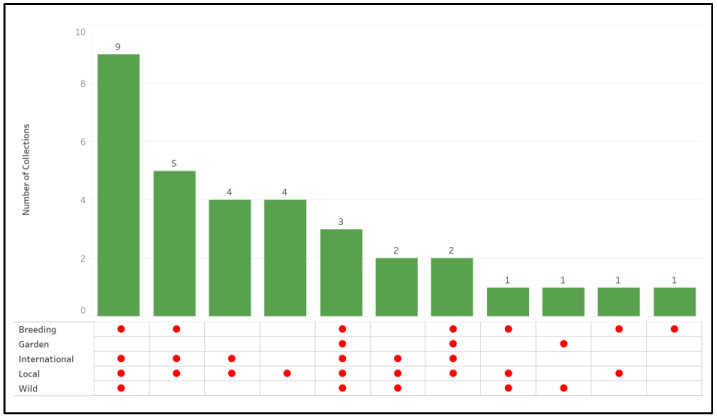
The conservation priorities of collections that hold various types of citrus plant genetic resources accessions.

The survey requested information about primary conservation priorities. Most collections focused on conserving breeding materials, international (widely available, commercial) and local (regionally available) cultivars, and wild species (wild undomesticated plants most closely related to agricultural crops). Fewer collections prioritized materials intended for gardens (Figure 2). 

**Figure 3:**
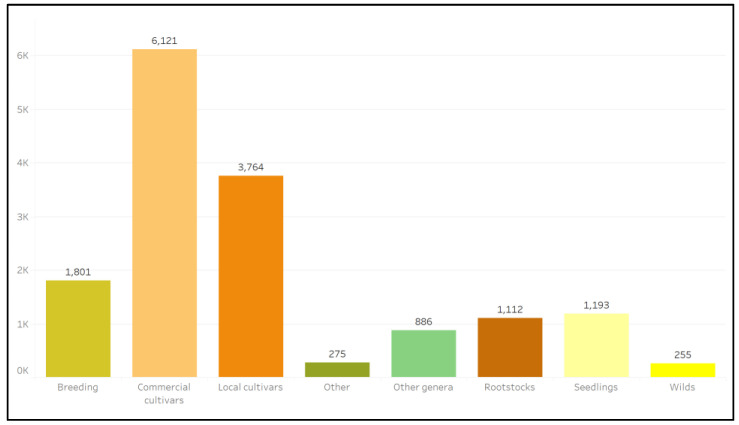
The number of ex situ citrus plant genetic resources reported in all survey responses that are classified in the listed categories.

Across the 33 respondents that provided this level of data granularity (Table S1), the highest number of genebank accessions were commercial and local cultivars, followed by materials for breeding, seedlings, rootstocks, and materials collected in the wild (Figure 3).

**Figure 4:**
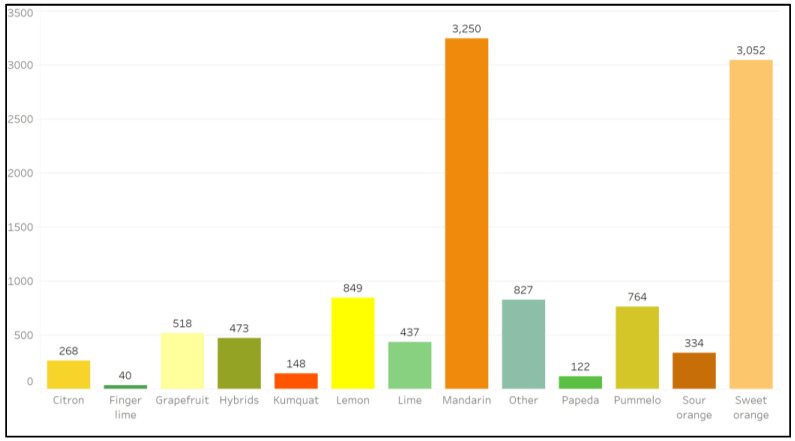
The number of ex situ citrus plant genetic resources reported in all survey responses that are classified as listed fruit types.

**Figure 5:**
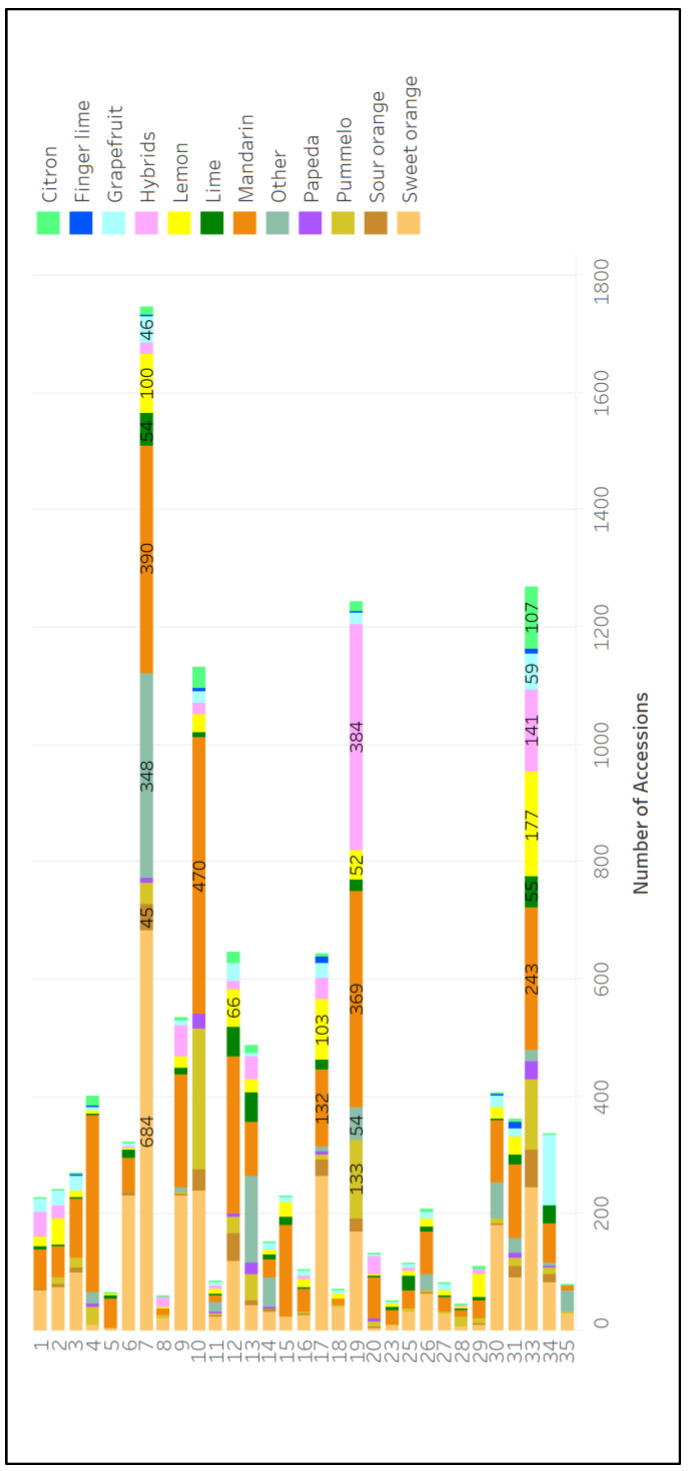
The number of accessions of each fruit type in each citrus collection (collection identity numbers correspond to the first column of Table S1).

Survey respondents were asked to classify citrus collection materials based on fruit types (mandarin, sweet orange, lemon, pummelo, grapefruit, hybrids, lime, sour orange, citron, kumquat, papeda, and finger lime); the results are summarized in Figure 4. The largest accession holdings across all genebanks are mandarins and sweet oranges (Figure 4). Specific collection compositions are shown in Figure 5 and Table S1. 

Citrus genebank collections can be maintained as field plantings, in pots within greenhouses and screenhouses, in tissue culture, as seeds in cold storage, or as cryopreserved shoot tips or seeds. The results from the survey revealed that citrus collections are primarily maintained as plants in the field or greenhouse/screenhouse. They are maintained as actively growing plant collections so that they are available for distribution (as budwood or entire plants), to ensure that the desired genotypes are maintained, and to avoid the extended juvenility period experienced when seeds are germinated. Citrus collections maintained in a clean state are mostly kept in protected environments. The two collections with significant in vitro components are the Federal Research Centre the Subtropical Scientific Centre of the Russian Academy of Sciences, Russia, and the Instituto Valenciano de Investigaciones Agrarias (IVIA), Spain. 

Citrus seeds are classified as having intermediate storage physiologies (tolerating some drying, but do not survive long-term in −20 °C freezer conditions) [26]. There are published protocols that describe optimum equilibrium relative humidities prior to storage [27,28]; in some cases, it may be necessary to remove seed coats or excise embryonic axes prior to dehydration [28]. Within the USDA-ARS (USA) citrus collection, seeds for rootstocks and virus indicators are collected, stored at 4 °C for up to 12 months, and distributed; long-term cryo-storage has not yet been implemented. EMBRAPA and INRAE also have some seed storage activities.

Duplicate plantings or cryopreserved back-ups improve the security of genebank collections [29,30]. The extent of duplicate plantings varies considerably. Some respondents stated that accessions are duplicated in other collections either within-country (Brazil, for example) or internationally. Some collections have a single tree in the field for each accession, others have a partial greenhouse duplication, many have 2–5 trees in the field or in greenhouse pots for each accession. For example, the USDA-ARS in Riverside, California, USA, in cooperation with the University of California Riverside, maintains two trees in the field and two trees in protective structures. Some collections rely on greenhouse backups of field collections, and IVIA maintains in vitro collection back-ups. Some collections cryopreserve recalcitrant seeds and/or embryos (INRAE and Indian Council of Agricultural Research) and over 400 USDA citrus accessions are cryopreserved as shoot tips by USDA-ARS. Barriers to collection back-ups include resources (funding, time), a lack of skilled workers, facilities, and orchard space, as well as some international intellectual property rights (IPR) for commercial cultivars. 

### 2.1. Collection Health

**Figure 6:**
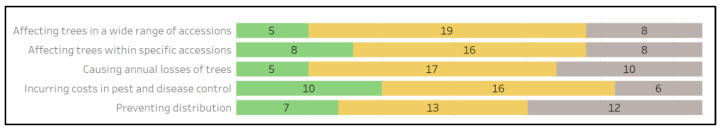
The number of collections that have major (green), minor (yellow), and no (gray) effects from pests and pathogens.

Citrus collection health is a serious concern. Some diseases, such as *Phytophthora-* caused root rot and CTV can be mostly managed using a combination of cultural (fungicide treatment) and genetic (tolerant/resistant rootstocks) practices. However, HLB poses a significant threat, with no current effective management options other than maintenance in protective structures. There are major collection threats due to pests and pathogens reported in as many as 10 collections. Listed scenarios included: “Affecting trees in a wide range of accessions”; “Affecting trees within specific accessions”; “Causing annual losses of trees”; “Incurring costs in pest and disease control”; and “Preventing distribution” (Figure 6). Citrus pests and diseases that threaten plant collections are listed in Table S2. The collections face different levels of pest and pathogen pressures because some collections have sufficient resources to maintain germplasm in protective structures with robust pest control, whereas others may lack the resources to do so. In addition, some collections are maintained in areas with greater pest or pathogen pressures, which would make control more difficult. Similarly, abiotic threats that may have an impact on maintenance (e.g., hurricanes, extreme temperatures), are greater at the locations of some genebanks than at others. Finally, collections without testing programs may be considered more robust health-wise than they actually are.

Survey responses revealed that tests are available for most threatening pests and pathogens, but many collections do not have the resources for regular testing. The extent of testing also varies widely with some collections performing no testing, others just testing for CTV and/or HLB, and others having comprehensive testing programs, such as IVIA, Citrus and Subtropical Fruits Research Center, Iran, and Citrus Research International, South Africa. Collections also vary with respect to if, and to what extent, they are maintained as cleaned-up plants. High percentages of cleaned-up plants are maintained by the following institutions: Embrapa Temperate Agriculture, Brazil (90%); INRAE, France (80%); IVIA (100%); Citrus Research International, South Africa (100%); Instituto Nacional de Tecnología Agropecuaria (INTA)—Concordia Experimental Station, Entre Ríos, Argentina (100%); Bodles Research Station Ministry of Agriculture and Fisheries, Jamaica (90%); and the Plant Resources Center, Vietnam (100%). 

Citrus has strict national and regional phytosanitary requirements that restrict the movement of propagation materials between, and sometimes within, countries. When budwood importation is allowed, quarantine regulations require isolation, testing, and clean-up (often shoot tip grafting) procedures [31]. Clean-up procedures currently focus on shoot tip grafting [32], whereas testing includes a range of laboratory- and biologically-based procedures [33]. These processes must be carried out in appropriate structures with proper safeguards in place [34]. Budwood can be transported across borders within Europe using a phytosanitary passport. Some countries have regulations in place that exempt budwood from accepted sources known to have high phytosanitary standards from some or all of the requirements. At the time of the survey, most citrus collections are not currently focused on filling collection gaps, although one collection also expressed interest in acquiring additional rootstocks for managing HLB. 

### 2.2. Collection Evaluation and Characterization

**Figure 7:**
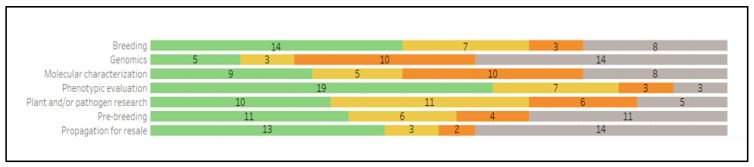
The number of collections that use materials on-site for listed purposes, and the frequency of those uses (frequent: green; moderate: yellow; rare: orange; never: gray).

Citrus collections are used on-site for phenotypic evaluation, breeding and pre-breeding, propagation for resale, and for plant and/or pathogen research. Collections were used for genomic characterization less frequently (Figure 7).

Most collections use the IPGRI Citrus descriptors for standardized phenotyping [35]. The methods for phenotyping are also published as internal manuals and on websites (China; Japan [36]) and within manuscripts [14,37,38,39,40,41,42,43]. Additional descriptor lists are published by the International Union for the New Varieties of Plants [44,45]. 

Citrus collections have been genotyped using a wide range of markers including RAPD [46,47], simple sequence repeats (SSR) [47,48,49,50,51,52,53], genotyping by sequencing (GBS) [54], and single nucleotide polymorphism (SNP) arrays (Queensland; CREA, unpublished [55]). Whole genome sequencing has been performed to assess genetic relationships and evolutionary history in citrus [4,56]. Phylogenetic analyses have also been performed using chloroplast sequence information [57,58].

### 2.3. Data Availability

Eleven of the 35 collections have databases with information (such as passport, taxonomy, images, phenotype, genotype, and/or pathogen status) that are, to some extent, available to the public (Table S3). These databases are mostly available in English and often also in other languages. Citrus genomic data, including some generated from materials in genebank collections, are available in publicly available external databases including The Citrus Genomic Variation Database (http://citgvd.cric.cn/home accessed on 8 February 2023) [59], the Citrus Pan-genome2breeding Database (http://citrus.hzau.edu.cn/index.php accessed on 8 February 2023) [60], and the Citrus Genome Database (https://www.citrusgenomedb.org/ accessed on 8 February 2023) [61]. Collections that do not have publicly available databases usually store information on local databases and spreadsheets. Some collections with publicly available databases also maintain local databases.

### 2.4. Collection Distribution

The Citrus Collection Survey asked respondents about the primary uses of their distributions. The most frequent uses are propagation for resale, certification programs, breeding, plant and/or pathogen research, phenotypic evaluation, and molecular characterization. To a lesser extent, collections are used for prebreeding and genomics. 

Most collections distribute materials for research purposes, and many materials are also distributed for breeding and commercial purposes. Sixteen collections distribute plant materials to the public. In total, about 3750 genebank accessions are distributed to about 350 users annually. Distributions from the large-scale nursery production of trees for commercial purposes (such as wholesale and retail production for industrial and nonindustrial uses) were not included in these counts. The survey did not ask users if DNA was stored and distributed. In the USDA, samples for molecular analyses are distributed as nucleic acids when it is possible to process the materials in a form that is acceptable to the user. 

Most citrus collections do not have limitations on material use, but there are some collections that only distribute for research (not commercial) purposes. Further, IPR restrictions may be in place and there are also some limitations on distributions to the public. Agreements (material transfer, cooperative, consortium agreements) may also be necessary. Most citrus collections distribute materials primarily within their countries without charge, or on a cost-recovery basis (including shipping and phytosanitary certification). The USDA-ARS and the Queensland Department of Agriculture and Fisheries are two collections that routinely distribute internationally. Some citrus collections sell propagated trees to fund conservation efforts. 

### 2.5. Resources

Survey respondents identified many ways in which collections could be improved. This included building new or renovating existing facilities, establishing and maintaining clean plants, increasing the number of trained staff, developing secure back-ups (which could involve cryopreservation), and making long-term commitments to genebank collections. 

Eleven collection managers, out of the 28 respondents to this question, stated that there is adequate retention of trained staff. Most collections are limited with respect to staffing; some collections only have a manager and some field personnel, and some do not have any dedicated personnel. Positions are vacant at some collections, and others rely upon students to maintain collections and acquire data. Many respondents stated that staff training is needed, particularly with respect to molecular characterization efforts. 

Overall, additional financial resources are needed for citrus collection management. Budgets range from reasonable institutional support to maintenance efforts supported from research grant proposals. There are capacity needs with respect to collection repropagation and greenhouse/screenhouse structure repair/replacement. About half of the collections responded that resource inadequacies will result in a loss of germplasm in collections. 

Survey responses listed several organizations that provide opportunities for networking at national, regional, and international levels. For example, India’s citrus programs are part of an All India Coordinated Research Project (AICRP) on fruits, the European Union has joint citrus projects, the Iberoamericana para la vigilancia de *Xylella fastidiosa* (IBER-SYFAS) is an Ibero-American effort focused on *Xylella*. The International Society of Citriculture, the International Society of Citrus Nurserymen, and the International Organization of Citrus Virologists, are international organizations with wide membership and interest in germplasm collections, to ensure the future availability of citrus genetic diversity. 

## 3. Discussion

Significant advancements have been made in ex situ citrus conservation since the time of Vavilov’s travels and observations. Citrus collections around the world conserve species and the cultivar diversity of *Citrus* and its crop wild relatives. Most citrus collections are maintained in vulnerable field or greenhouse/screenhouse conditions without adequate forms of secure back-ups. Collections are threatened by pests and pathogens as well as changing institutional financial obligations. Given the significant international, and even some within-country barriers to citrus movement, as well as the vulnerabilities of individual collections, it is important that citrus genetic resources be maintained in multiple genebanks around the world. In the future, collection genotyping and phenotyping efforts will reveal the genetic relationships within and among collections, and further inform prudent conservation efforts.

Despite the importance of citrus collections for future breeding and production, there are no global working groups focused on the conservation of citrus genetic resources at this time. The survey results presented herein provide a first step toward identifying international collections that could be included in a global citrus conservation network. Despite extensive efforts to identify and contact collections, the list of survey responses is not comprehensive. This is due to several factors, including language barriers, out of date contact information in databases, new genebank managers, and because individual citrus collections may be isolated and not integrated into larger genebanks with an identifiable web presence. 

“A Global Strategy for the Conservation and Use of Citrus Genetic Resources” has been drafted and will be posted on the website of the Global Crop Diversity Trust [22]. This strategy document includes a wide-ranging review of citrus taxonomy, crop wild relatives and domestication, aspects of collection management, as well as breeding opportunities. In addition, it includes specific tabular and graphical information from the survey described herein, as well as a set of priority actions that would help strengthen the global citrus community and improve the conservation of this important crop.

In conclusion, this survey is an initial step towards identifying and documenting citrus genebank collections around the world. The Global Strategy for the Conservation and Use of Citrus Genetic Resources proposes six priority actions that will help unify the community. These include: the development of an international working group to guide the development of a Citrus Community Information System; to support data collection and documentation efforts for citrus collections; to identify and fill taxonomic gaps in collections; to increase citrus collection health and security; to provide training opportunities; and ultimately to develop, maintain and distribute clean, secure, citrus genetic resources from a diverse international collection [22]. Additional community building and networking could provide opportunities for new research partnerships, and collaborative funding opportunities.

## 4. Materials and Methods

In 2021, a survey was developed using the SurveyMonkey Audience platform (www.surveymonkey.com/mp/audience 9 February 2023) and widely distributed within the citrus genebanking community. Surveys were distributed to collection contacts identified by personal sources, journal article authors, the Genesys database (https://www.genesys-pgr.org/ 9 February 2023), and FAO WIEWS (https://www.fao.org/wiews/en/ 9 February 2023) [24,25]. The survey requested information about: the composition; ex situ management; data collected and its availability; health; security back-up; human resources, distribution and use; policies; and the future development of citrus collections. Follow up reminders were sent to ask contributors to complete surveys. The survey results were downloaded, and duplicate submissions were removed.

A total of 43 unique survey responses were received from a wide range of genebanks in 27 countries. Although breeding collections were not specifically targeted, some responses from breeding collections were received. Inventory data for seedling populations in breeding collections were not included in the tabulations. Of the 43 survey responses received, some collection responses from Cambodia, Taiwan, Thailand, Turkey, India, and Vietnam, were limited to contact information and consequently were not included in further analyses. Citrus organizations that provide clean plant materials to local citrus nursery industries for the commercial production of trees were not included. These included: AusCitrus, (the Australian Citrus Propagation Association Incorporated), and the California Citrus Clonal Protection Program and Florida Department of Agriculture and Consumer Services Bureau of Citrus Budwood Registration, in the United States. Sections of some submitted survey responses were incomplete and therefore were not included in the analyses for those sections.

## Data Availability

Data are provided in supplementary tables.

## Supplementary Materials

The following supporting information can be downloaded at: https://www.mdpi.com/article/10.3390/plants12040814/s1, Table S1: Citrus collections and their composition described by survey respondents; Table S2: Diseases and pathogens that are present and threaten collections; Table S3: Citrus collections with databases available to the public.
